# Electrocardiographic abnormalities in patients with cardiomyopathies

**DOI:** 10.1007/s10741-023-10358-7

**Published:** 2023-10-17

**Authors:** Alberto Aimo, Agnese Milandri, Andrea Barison, Andrea Pezzato, Paolo Morfino, Giuseppe Vergaro, Marco Merlo, Alessia Argirò, Iacopo Olivotto, Michele Emdin, Gherardo Finocchiaro, Gianfranco Sinagra, Perry Elliott, Claudio Rapezzi

**Affiliations:** 1 Interdisciplinary Center for Health Sciences, Scuola Superiore Sant’Anna, Pisa, Italy; 2 Cardiology Division, Fondazione Toscana Gabriele Monasterio, Pisa, Italy; 3 Cardiology Unit, Bentivoglio Hospital, Bologna, Italy; 4 Center for Diagnosis and Management of Cardiomyopathies, Cardiothoracovascular Department Azienda Sanitaria Universitaria Giuliano Isontina (ASUGI) and University of Trieste, Trieste, Italy; 5 Careggi University Hospital, Florence, Italy; 6 Department of Experimental and Clinical Medicine, University of Florence, Meyer Children Hospital Florence, Florence, Italy; 7 Royal Brompton and Harefield Hospital, London, UK; 8 St George’s University of London, London, UK; 9 UCL Centre for Heart Muscle Disease and Lead of the Inherited Cardiovascular Disease Unit, Bart’s Heart Centre, London, UK; 10 Cardiology Centre, University of Ferrara, Ferrara, Italy

**Keywords:** ECG, Electrocardiogram, Cardiomyopathy, Hypertrophic cardiomyopathy, Amyloidosis

## Abstract

Abnormalities in impulse generation and transmission are among the first signs of cardiac remodeling in cardiomyopathies. Accordingly, 12-lead electrocardiogram (ECG) of patients with cardiomyopathies may show multiple abnormalities. Some findings are suggestive of specific disorders, such as the discrepancy between QRS voltages and left ventricular (LV) mass for cardiac amyloidosis or the inverted T waves in the right precordial leads for arrhythmogenic cardiomyopathy. Other findings are less sensitive and/or specific, but may orient toward a specific diagnosis in a patient with a specific phenotype, such as an increased LV wall thickness or a dilated LV. A “cardiomyopathy-oriented” mindset to ECG reading is important to detect the possible signs of an underlying cardiomyopathy and to interpret correctly the meaning of these alterations, which differs in patients with cardiomyopathies or other conditions.

**Graphical abstract:**

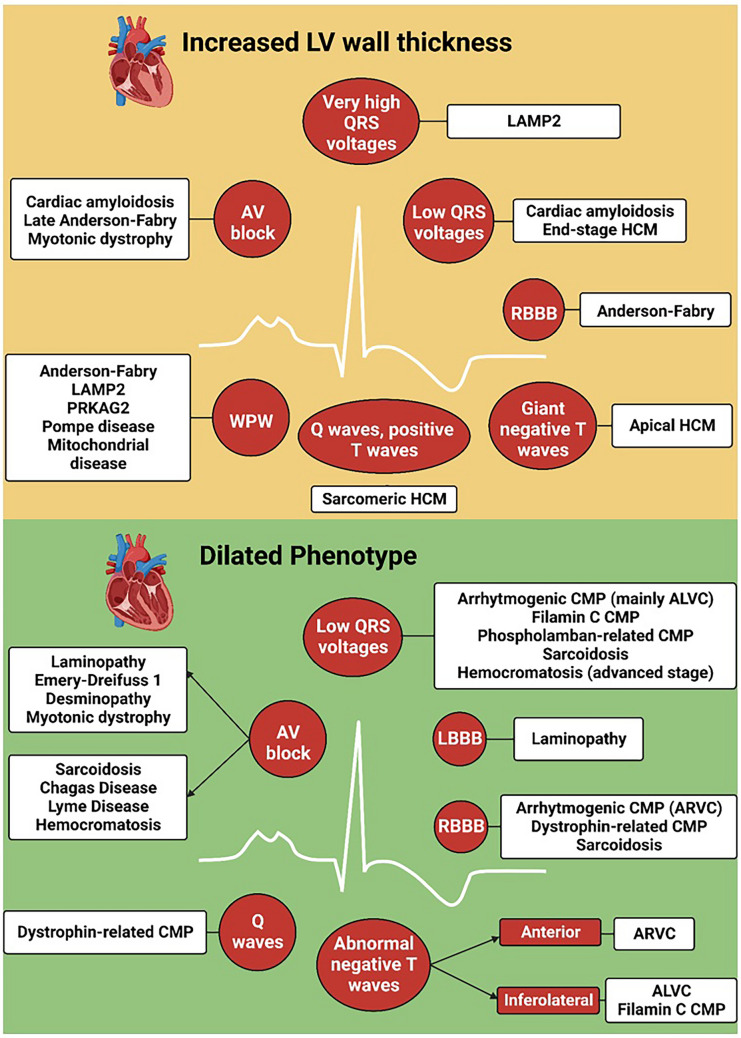

**Supplementary Information:**

The online version contains supplementary material available at 10.1007/s10741-023-10358-7.

## Introduction

Abnormalities in impulse generation and transmission are among the first signs of cardiac remodeling in cardiomyopathies. These abnormalities manifest as changes on the 12-lead electrocardiogram (ECG). The ECG may be a source of red flags for diagnosis, may help to define the disease stage and predict patient outcomes, and sometimes even suggest specific genetic backgrounds. Herein, we will provide an overview of the ECG findings in patients with cardiomyopathies and their clinical significance. We will focus on ECG findings in adult patients, as cardiomyopathies in children would require a dedicated paper. The following “cardiomyopathy-oriented” approach to ECG will refer to specific diseases more than the large nosographic categories proposed by new Guidelines [1]. Most notably, the non-dilated left ventricular (LV) cardiomyopathy will not be considered as a clinical entity, to better characterize its various manifestations (e.g., laminopathies, neuromuscular disease, and sarcoidosis). Furthermore, LV non-compaction (LVNC) will be still regarded as a specific disorder rather than as a “hypertrabeculated” phenotype associated with other forms of cardiomyopathy, as recently proposed [1].

## P wave and PR interval

### P wave abnormalities

Structural atrial disease may alter P wave duration or morphology. Patients with hypertrophic cardiomyopathy (HCM) have longer P wave duration (92% of cases) and greater P wave dispersion than healthy subjects [2]. P wave duration was associated with a more severe HCM phenotype and left atrial (LA) electromechanical delay, while P wave dispersion with a more severe diastolic dysfunction and mitral regurgitation [2]. Among patients with dilated cardiomyopathy (DCM), 72% had signs of LA enlargement and 15% of right atrial (RA) enlargement [3].

### Long PR interval and atrioventricular blocks

Expansion of extracellular spaces or the accumulation of intracellular material may lead to a prolongation of PR interval and atrioventricular (AV) blocks.

In cardiac amyloidosis (CA), the ECG can show first- (21%), second-, or third-degree AV block (3%) [4]. The incidence of symptomatic AV block is higher in amyloid transthyretin (ATTR)—than light-chain (AL) CA (first-degree AV block was present in 18% of those with AL-CA, but up to 33% of those with ATTRwt-CA and 25% for ATTRv [5], possibly because these patients are older and have a greater amyloid burden and longer survival.

The cardiac phenotype of neuromuscular diseases is highly variable. Cardiac involvement is found in 80% of patients with myotonic dystrophy (DM) type 1 and 10–20% of patients with DM type 2. Furthermore, up to 20% of patients with DM type 1 develop progressive AV or intraventricular conduction defects and ventricular or supraventricular arrhythmias [6]. Conduction defects in DM type 2 are usually limited to first-degree AV or bundle branch block (BBB), but life-threatening arrhythmias and sudden cardiac death (SCD) have been reported [7]. Conduction defects ranging from sinus bradycardia, prolongation of the PR interval, to complete heart block can also be detected in Emery-Dreifuss cardiomyopathy [8].

Laminopathies are caused by *LMNA* gene mutations. Up to 92% of patients > 30 years have arrhythmias, including ventricular ectopic beats, non-sustained ventricular tachycardia, and first-degree AV block (which may progress to more advanced AV blocks) [9]. AV block is due to fibrosis in the AV node. Even asymptomatic mutation carriers with preserved or only mildly decreased LV contractility or patients with minor conduction defects have a high risk of SCD [10].

In cardiac sarcoidosis (CS), AV blocks may develop because of granulomas affecting the conduction system; in a prospective study on middle-aged patients with unexplained AV block, 34% had CS [11]. Iron chelating therapy has substantially reduced the risk of AV blocks in patients with hemochromatosis [12]. First-degree and advanced AV block are found in 3–25% of patients with LVNC) [13].

### Short PR interval

A PR interval < 120 ms may denote accelerated conduction through the AV node or accessory pathways. Disease progression may lead to the development of AV blocks.

Anderson-Fabry disease (AFD) is characterized by glycosphingolipid accumulation increasing conduction velocity. AFD should be suspected in patients with LV hypertrophy (LVH) and a short PR interval. Similarly, a short PR interval, prolonged QRS duration, right BBB (RBBB), R in aVL ≥ 1.1 mV, and ST depression in the inferior leads may help differentiate AFD from HCM [14]. Progressive glycosphingolipid deposition can ultimately lead to AV blocks. First-degree AV block disappearance following enzyme replacement therapy (ERT) was reported [15].

Several studies reported abnormalities within the AV node and His bundle in glycogen storage disorders, including the presence of fasciculoventricular pathways possibly due to disruption of the physiological conduction because of glycogen-filled myocytes [16]. Danon disease is due to deficient lysosome-associated membrane protein 2. A pattern of ventricular pre-excitation is found in over 70% of cases. These patients have also a high risk of atrial and ventricular arrhythmias and SCD even at a young age [17]. Pompe disease is an autosomal recessive disease caused by a deficiency in the lysosomal enzyme alpha-1, 4 glucosidase. LVH is found in 12% of patients and a short PR interval in 10% [18]. ERT leads to increased PR interval and decreased LV voltages in children [19], but not in adults [18]. PRKAG2 syndrome is caused by abnormalities in the Ras/MAPK pathway causing glycogen accumulation within the cardiomyocytes. The most common ECG findings are a short PR interval (in 68% of patients), a BBB (mainly RBBB), abnormal QRS morphology, intraventricular conduction delays > 120 ms, and, in the later disease stages, advanced AV or sinoatrial blocks [20]. These patients have a higher risk of SCD, probably due to high-degree AV block or fast conduction of supraventricular tachyarrhythmias through accessory pathways [20].

Mitochondrial diseases are due to mitochondrial or nuclear DNA mutations. In a small cohort, 68% had an abnormal ECG, and 22% of them presented a pre-excitation pattern [21]. AV block is common in Kearns-Sayre syndrome, possibly because of a higher mutation load in the AV node [22].

## Atrial fibrillation

Atrial fibrillation (AF) often complicates the course of inherited cardiomyopathies and may be the presenting feature [23].

AF is found in 17 to 30% of patients with HCM, most commonly in elderly patients and those with LV outflow obstruction [24, 25]. Atrial enlargement and fibrosis are associated with a higher risk of AF [23, 26]. Patients with HCM and AF have higher risk of all-cause mortality and cardiac deaths compared to HCM controls without AF, with an increased incidence of SCD and HF- and stroke-related death [27].

The prevalence of AF in familial DCM ranges between 36 and 76% [23, 24, 28–30]. The prevalence of AF in familial DCM is close to the prevalence of non-familial form [31, 32].

Patients with right-sided arrhythmogenic cardiomyopathy (ACM) show AF in 9 to 30% of cases [33, 34]. AF is likely a consequence of atrial involvement due to desmosomal dysfunction, RA enlargement and dysfunction, or both. Atrial conduction abnormalities have been found in right-sided ACM, resulting in P wave alterations independent from RV morphological anomalies [35]. Furthermore, AF predicts worse outcomes in patients with right-sided ACM [36].

AF has been reported in 1 to 29% of patients with LVNC, with a lower incidence in children than in adults [23, 24, 37, 38]. AF is associated with LVNC severity and predicts survival [39, 40].

The prevalence of AF in patients with CA ranges between 15 and 44% and is higher in ATTR-than AL-CA. Cardiac amyloid infiltration causes ventricular wall thickening and diastolic dysfunction, leading to atrial dilation and predisposing to AF [41, 42]. Amyloid deposition promotes atrial fibrosis and remodeling, further increasing the risk of AF [43]. AF does not seem to predict a worse outcome [44, 45].

## Q waves

**Fig. 1:**
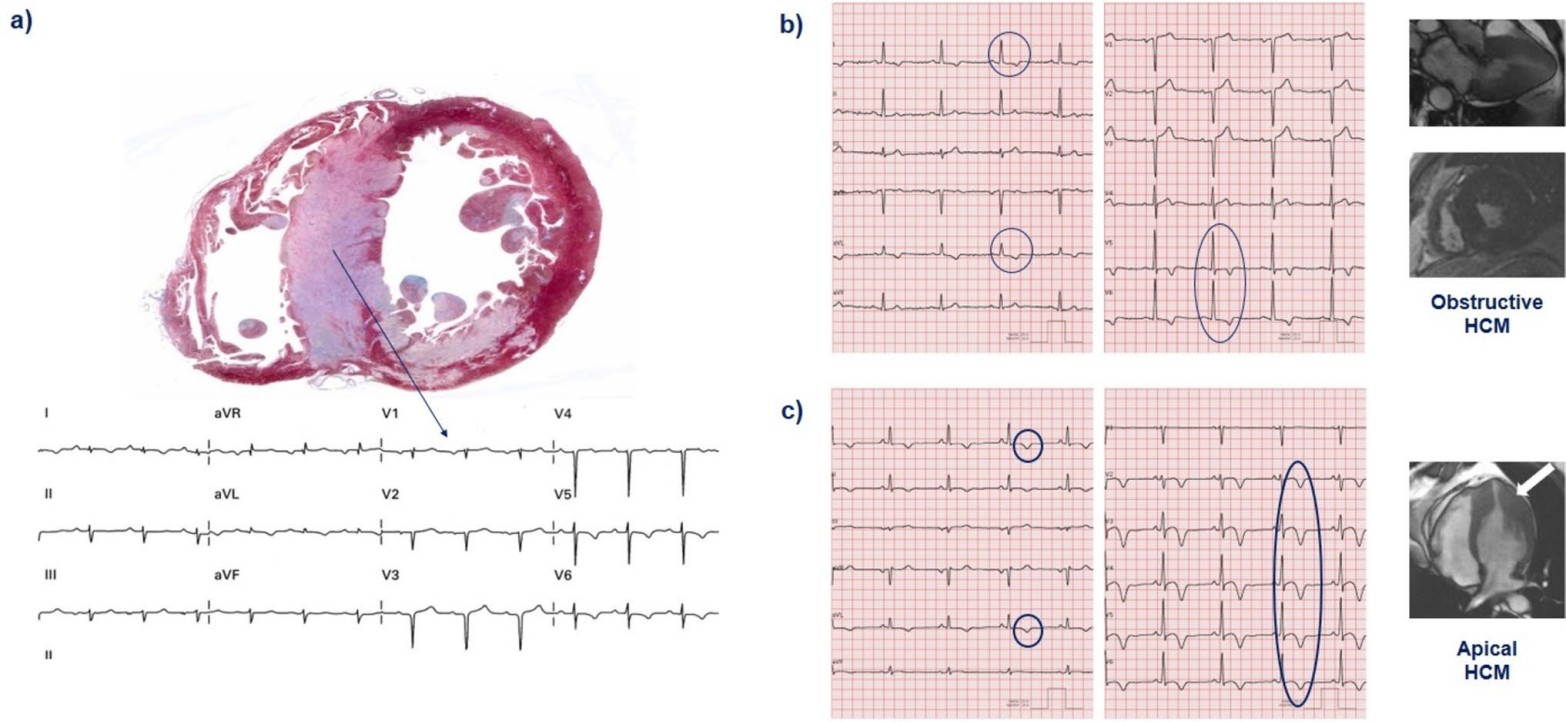
Possible electrocardiographic findings in patients with left ventricular hypertrophy. **a** 72-year-old man with transthyretin amyloidosis experiencing sudden cardiac death. The explanted heart shows massive septal hypertrophy, explaining the poor R wave progression and QS complexes in V2–V3. Negative T waves in the antero-lateral leads: **b** 55-year-old man with obstructive hypertrophic cardiomyopathy (HCM) and **c** 49-year-old man with apical HCM

Q waves are found in 18 to 53% of patients with HCM [46]. The possible mechanisms are a loss of local electrical forces due to transmural fibrosis (Fig. 1a) or an initial displacement of the QRS vector due to a disproportionate thickening of the basal interventricular septum and/or basal LV free wall. Q waves may precede LV mass increase by several years and are less common in patients with biventricular hypertrophy [47].

Q waves in DCM reflect vector displacement due to LV dilation and transmural fibrosis and were reported in 10 to 25% of patients [3, 48].

Q waves in CA are likely associated with the accumulation of amyloid and fibrosis. CA should be suspected when Q waves, particularly in the anterior leads, coexist with low QRS voltages or there is a discrepancy between QRS voltages and LV mass. Q waves seem to be more common in patients with AL-CA, being reported in 25 [49] to 47% [50], than in ATTR-CA (18%) [49].

## QRS complex: high voltages

More than 30 ECG criteria to diagnose LVH have been developed, which are neither sensitive nor specific to detect LVH [51], particularly in cardiomyopathies, where the mechanism of increased LV mass is often different from the expansion of conductive tissues. ECG criteria for RV hypertrophy (RVH) have been proposed, but have poor sensitivity, likely because, for RVH to be manifested on the ECG, it must be severe enough to overcome the concealing effects of the larger LV forces [52]. ECG criteria for LVH are met by 41 [53] to 60% [46] of patients with HCM (Fig. 1b). Interestingly, LVH patterns such as increased voltages in the precordial leads and deep Q waves may precede LV mass increase in mutation carriers, possibly reflecting myocardial disarray, interstitial fibrosis, and microvascular remodeling [54]. In patients with established HCM, a single positive criterion for LVH is very rare. ECG criteria for LVH hold prognostic significance, and a score including QRS amplitude was proposed to predict SCD [55]. Anecdotal experiences suggest that a 6-week therapy with the myosin inhibitor mavacamten relieves ECG signs of LVH (Supplemental Fig. 1), possibly reflecting a restoration of the electrical properties of cardiomyocytes.

ECG criteria for LVH are met in 17 to 69% of patients with DCM [56, 57]. LVH is infrequent in ACM because of the progressive fibro-fatty replacement of the myocardium which often results in low QRS voltages on the 12-lead ECG.

Patients with CA have usually no ECG features of LVH, but normal or increased ECG voltages can be found in up to 25% of patients with ATTR-CA. The LV apex and the periapical segments are relatively spared by amyloid accumulation and may still generate sufficiently high voltages in leads V3 and V4 to meet ECG criteria for LVH [58].

ECG features of LVH are rare in some storage disorders (e.g., 6–10% in Pompe disease) [18, 19] and more common in others, such as Danon and Fabry disease, because of increased LV electrical mass due to glycosphingolipid accumulation driving cell enlargement [59]. Patients with Danon disease often exhibit particularly prominent QRS complexes [59]. ECG voltage criteria of left or biventricular hypertrophy are detected in > 40% of patients with LVNC [13].

## QRS complex: low voltages

The most common definition of low QRS voltages is a nadir-to-zenith QRS amplitude in all peripheral leads ≤ 0.5 mV and ≤ 1 mV in all precordial leads. Low QRS voltages may be associated with cardiomyocyte loss and/or expansion of extracellular spaces by electrically inert tissue such as fibrosis, fat, or amyloid. Low QRS voltages, including cases where this feature is isolated, may be an expression of early-stage cardiomyopathy deserving further investigation [60]. Low QRS voltages may also be due to pericardial effusion or extra-cardiac conditions such as obesity or emphysema.

Low QRS voltages are rare in patients with HCM (< 3%) and can be seen in some patients with end-stage disease due to the extensive fibrosis [61]. Low QRS voltages predict SCD [61], but not a combination of all-cause death, major non-fatal arrhythmias, hospitalization for heart failure (HF), and stroke after adjustment for the main demographic and clinical variables [62].

Only 3–6% of patients with DCM have low QRS voltages and about 1.5% in both precordial and peripheral leads [3]. Loss of vital myocardium and diffuse LV fibrosis may reduce QRS amplitude, especially in precordial leads [3].

Low QRS voltages in the limb leads are found in 41% of patients with ACM and LV involvement and just in 17% of those without LV involvement [63]. Several studies showed a higher prevalence of low QRS voltages in ACM than in idiopathic RV outflow tract tachycardia [64], athlete’s heart [65], or DCM [63, 66]. The association between low QRS voltages and the extent of fibro-fatty replacement of the LV accounts for the prognostic significance of this finding [67].

Low QRS voltages in peripheral leads are found in 46 to 70% of patients with CA [68]. Low QRS voltages are found more often in patients with AL-CA than in ATTR-CA, but are a risk factor for cardiovascular death in both conditions [69]. As the ventricles are more affected, low QRS voltages may be associated with normal P wave voltages on peripheral leads. Low QRS voltage is also a common finding in non-dilated LV cardiomyopathy caused by *DSP* (15–44%) and *PLN* (15%) mutations [70–72].

## QRS fragmentation and epsilon wave

QRS fragmentation (defined as the presence of various RSR′ patterns, R or S notching, and/or > 1 additional R wave in any non-aVR lead) is due to heterogenous action potential propagation different from BBB, due to focal alterations such as fibrosis or fibro-fatty replacement.

QRS fragmentation was detected in 75% of patients with HCM [73]. QRS fragmentation in ≥ 3 territories (inferior, lateral, septal, and/or anterior) had an incremental risk of ventricular tachyarrhythmias and SCD beyond conventional risk factors [73]. In DCM, QRS fragmentation has been reported in 23–26% of patients and predicts ventricular tachycardia and all-cause mortality [74], reasonably because of its relationship with the extent of LV fibrosis. In LVNC, the presence of fragmented QRS was associated with a significantly lower survival rate [75].

The epsilon wave is a low-voltage deflection between the QRS complex and the ST segment in the right precordial leads (V1–V3) (Supplemental Fig. 2). It is produced by a delayed RV free wall activation in the subepicardial regions due to fibro-fatty replacement. Epsilon waves in right precordial leads are currently classified as minor diagnostic criteria for ACM with RV involvement [66]. Epsilon waves in inferior or lateral peripheral leads may signal advanced stage or LV involvement. Diffuse epsilon waves, particularly if present in aVR, are related to low RV ejection fraction, high rate of HF hospitalization, HF-related death, SCD, and heart transplantation [76]. QRS fragmentation is more sensitive than the epsilon wave (present in about 10–35% of patients with ARVC) to diagnose ACM, but is less specific [77]. QRS fragmentation predicts a higher arrhythmic risk [78].

**Fig. 2:**
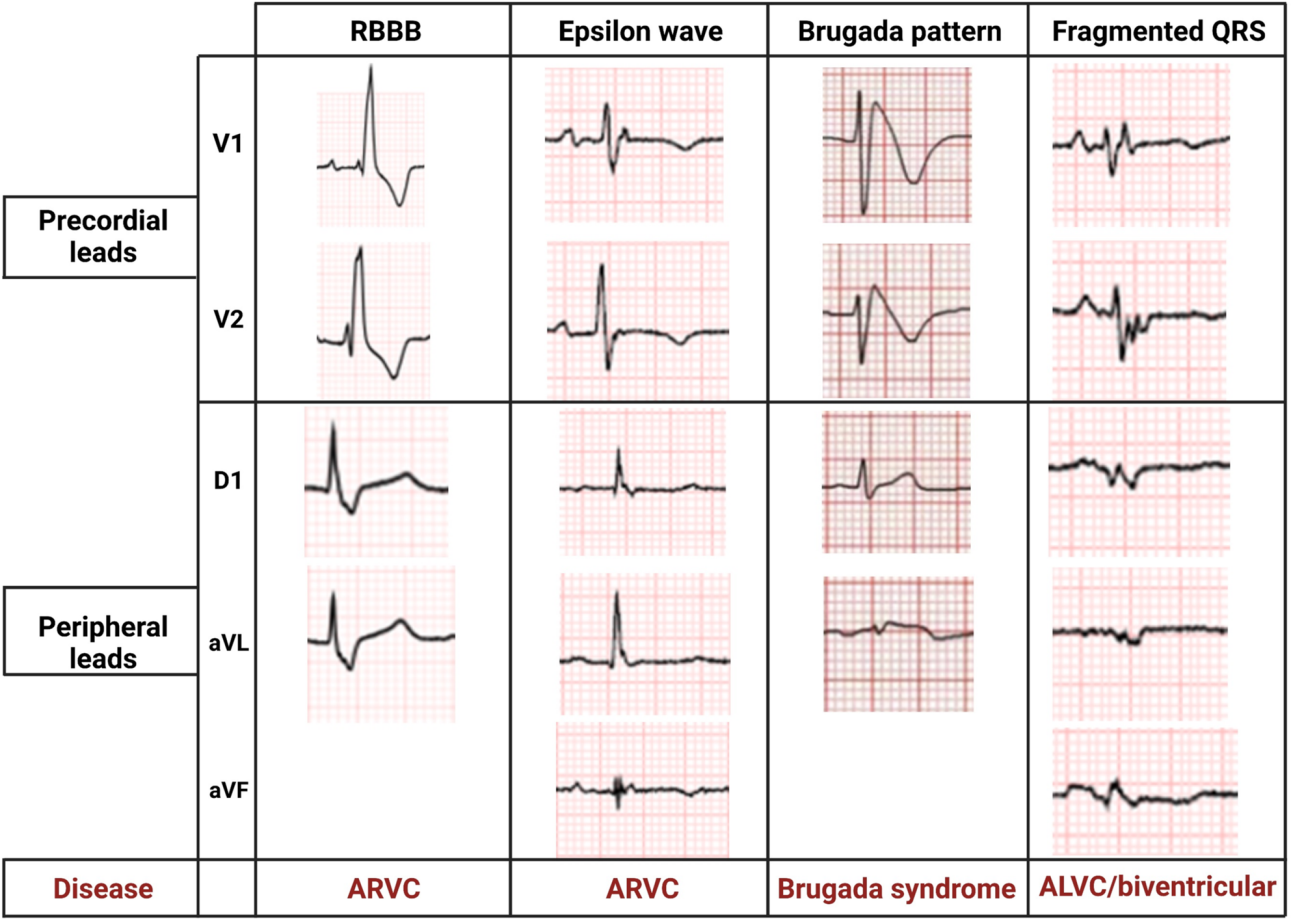
Right bundle branch block and its mimics. ALVC/ARVC, arrhythmogenic left/right ventricular cardiomyopathy

## Bundle branch blocks

Figure 2 provides some examples of RBBB and its possible mimics (fragmented QRS, epsilon wave, and Brugada pattern). The RBBB was reported in 4–5% of patients with HCM [79] and in 7% of patients with DCM [80]. A complete or incomplete RBBB was found in 32% of patients with right-sided ACM (*n* = 100) [81], while a larger study (*n* = 374) reported a prevalence of 19% [82]. RBBB may conceal depolarization abnormalities such as epsilon wave in V1 and V2 and QRS fragmentation in V1 making the diagnosis of ACM more challenging.

Left BBB (LBBB) is an uncommon finding (detected in 2%) of patients with HCM [83], mainly caused by the contractive asynchrony due to degeneration or fibrotic infiltration of the conduction system, with higher prevalence (22%) in end-stage disease reflecting a severe impairment of LV conduction [84]. LBBB may develop in up to 40% of cases after septal myectomy following damage to LBBB, but has no clear impact on mortality [79].

LBBB is common in patients with DCM, with a reported prevalence of 25–30% [86, 87]. LBBB does not predict mortality in patients with DCM and LV ejection fraction between 36 and 50% [87], while LBBB development independently predicts mortality in patients with idiopathic DCM, suggesting the possible benefit of early cardiac resynchronization therapy [88].

## ST segment and T wave

T wave inversion is frequent in HCM (Fig. 1b). Epicardial cardiomyocytes depolarize and repolarize later than endocardial cardiomyocytes, creating a ventricular repolarization vector in the opposite direction from the QRS. Symmetric negative T waves are common in inferolateral leads. Negative T waves > 0.1 mV in all antero-lateral precordial leads suggest apical hypertrophy [89] (Fig. 1c) and could precede the development of LVH detectable at echocardiography or cardiac magnetic resonance. Giant symmetric positive T waves in the precordial leads may be an early manifestation of HCM and could be associated with persistent ST-segment elevation. ST-segment elevation myocardial infarction patterns (ST-segment segment elevation without giant positive T waves, giant positive T waves without ST segment elevation, or both ST segment elevation and giant T waves) are all independently associated with the risk of SCD in HCM [61]. ST-segment depression > 0.2 mV plus T wave inversion in precordial leads and ST-segment depression in high lateral leads (DI-aVL) were also associated with SCD [61].

T wave inversion in the anterior and right precordial leads in individuals with complete pubertal development (in the absence of complete RBBB) are major diagnostic criteria for right-sided ACM, while T wave inversion in leads V1 and V2 only or inverted T waves in V1–V3 and V4 in adult individuals with complete RBBB are minor diagnostic criteria [66]. T wave inversion is caused by fibro-fatty infiltration beginning in the subepicardium, where depolarization and repolarization become more delayed compared to the subendocardium. The J-point preceding the anterior T wave inversion could differentiate between athlete’s heart physiological adaptation and cardiomyopathy [90]. The extent of T wave inversion is related to the amount of fibro-fatty infiltration. Negative T wave depth ≥ 0.2 mV in V1 is strongly related to disease presence with a high negative predictive value [91]. Downsloping ST segment elevation with negative T wave in V1–V2 is related to advanced transmural right ventricular involvement, and negative T waves in inferolateral leads are common in left-sided ACM [92].

AFD can present with asymmetrical negative T waves and ST-T segment depression or elevation in inferolateral leads, and their presence seems related to fibrosis. Finally, CA is often characterized by non-specific repolarization abnormalities such as flattened or shallow T waves. Corrected QT interval prolongation is common in patients with LVNC (> 440 ms in 38%), but its significance is unclear [13].

## Importance of a “cardiomyopathy-oriented approach” to ECG reading

**Fig. 3:**
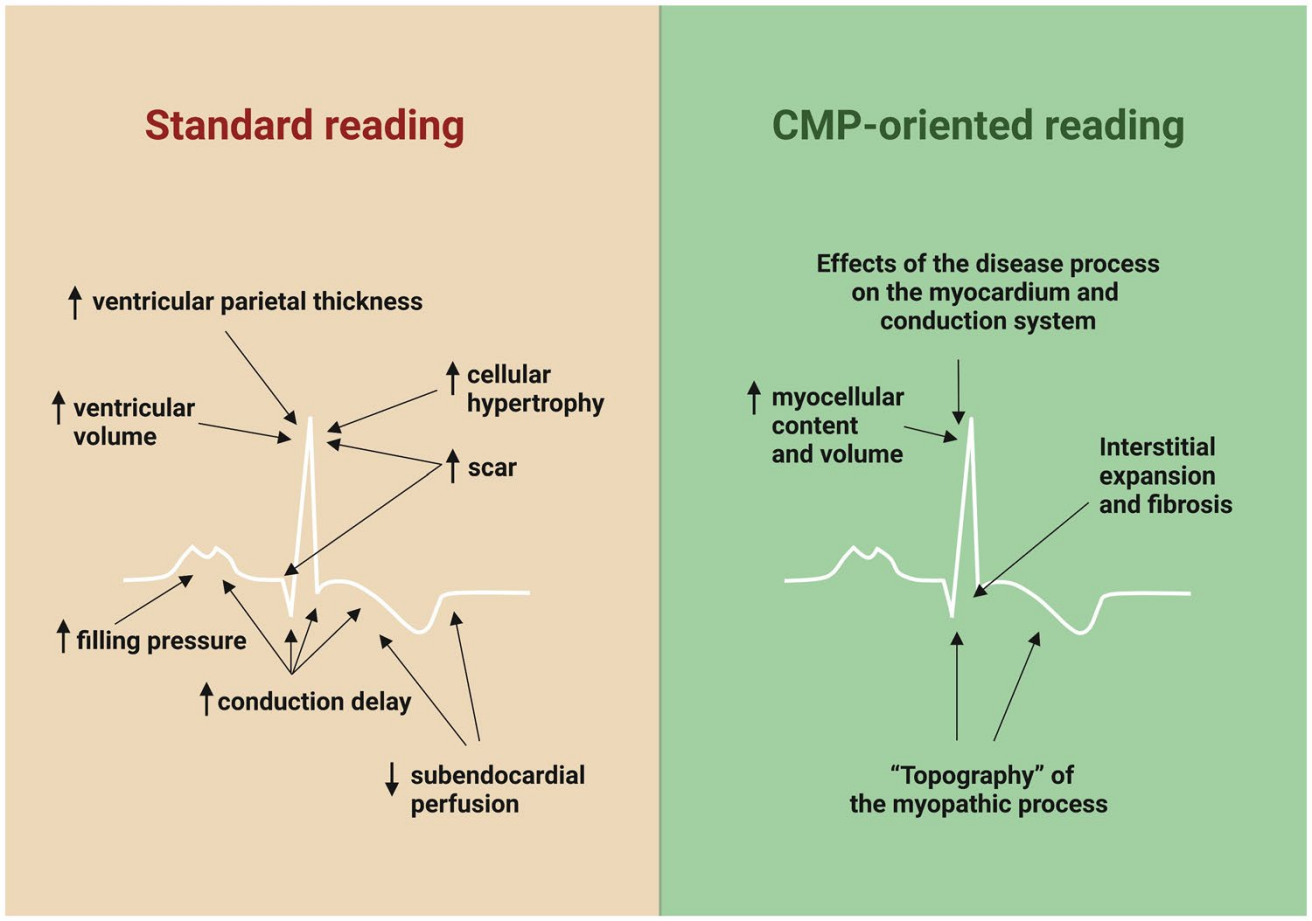
Standard vs. cardiomyopathy (CMP)-oriented approach to electrocardiogram reading. Central Illustration Electrocardiographic findings in patients with a hypertrophic or dilated phenotype. ALVC/ARVC, arrhythmogenic left/right ventricular cardiomyopathy; AV, atrio-ventricular; CMP, cardiomyopathy; HCM, hypertrophic cardiomyopathy; LAMP2, lysosomal-associated membrane protein 2; LBBB, left bundle branch block; PRKAG2, protein kinase AMP-activated non-catalytic subunit gamma 2; RBBB, right bundle branch block; WPW, Wolff-Parkinson-White

Patients with cardiomyopathies can display multiple ECG abnormalities (Table 1). A “cardiomyopathy-oriented” approach to ECG reading is important to detect the possible signs of an underlying cardiomyopathy and to interpret correctly these abnormalities (Fig. 3). Some findings are specific for certain disorders, such as the discrepancy between QRS voltages and LV mass for CA or the inverted T waves in the right precordial leads for ACM. Other findings are less specific, but may orient toward a specific diagnosis in patients with a hypertrophic or dilated phenotype (Central Illustration). Our understanding of the significance of ECG findings could be improved by studies aimed at correlating ECG with imaging or electrophysiological features, genetic background, and outcome. Another intriguing perspective is the automated interpretation of artificial intelligence (AI), which could assist clinicians in diagnosis, risk stratification, and follow-up. A great effort is needed to assemble large-scale datasets of digital ECG tracings. For diagnostic purposes, these datasets should include patients with a given cardiomyopathy and control individuals, who should be matched as closely as possible to patients (i.e., patients evaluated for a suspicion of the same cardiomyopathy, which was ultimately ruled out), rather than healthy individuals retrieved by institutional registries or patients with other cardiomyopathies. Properly trained AI algorithms could also capture subtle features that may inform on the response to treatment and disease evolution. Overall, the ECG still provides pivotal diagnostic and prognostic information and will probably acquire an even more important role in association with modern technologies such as AI.

**Table 1:** ECG findings, their pathogenesis, and meaning in cardiomyopathies

***ECG findings***				
***Disease***	***Pathogenesis/electrogenesis***	***Prevalence***	***Disease stage***	***Prognostic/therapeutic implications***
**P wave**				
**HCM**	LA and/or RA dilation	92% [2]		P wave prolongation associated with the severity of HCM phenotype [2]
**DCM**	LA and/or RA dilation	72% (if LA dilatation) 15% (if RA dilatation) [3]		
**Long PR interval and AV blocks**				
**AFD**	Accumulation of glycosphingolipids	6–11% [93]		First-degree AV block may disappear following ERT [15]
**CA**	Accumulation of amyloid and fibrous tissue	21% (1^st^ degree), 3% (2^nd^–3^rd^ degree) [4] Higher prevalence in ATTR-CA (1^st^ degree AV block in 18% patients with AL-CA but up to 33% of those with ATTRwt-CA and 25% for ATTRv) [5]		Same indications to PM implantation than in other disease settings [94]
**KSS**	Greater mutation load in AV node	84% [95]		High risk of complete AV block and SCD [94]
**Neuromuscular diseases:** **Emery-Dreifuss** **DM type 1** **DM type 2**		Up to 20% [96]		‐ Emery-Dreifuss dystrophy: risk of AV block and SCD [97] ‐ DM type 1: risk of AV block and SCD [6] ‐ DM type 2 usually leads to mild AV or BBB, but arrhythmias and SCD have been reported [7]
**Cardiac sarcoidosis**	Granulomatous inflammation affecting the conduction system	34% among middle-aged patients with unexplained AV block [11]		
**Laminopathy**	Fibrosis in the AV node		Early	Higher risk of life-threatening arrhythmias [9]
**LVNC**		3–25% [13]		
**Short PR**				
**Danon disease**	Accelerated nodal conduction, disruption of the annulus fibrosus by glycogen-filled myocytes	70% in M [17]		Risk of lethal arrhythmias and SCD following atrial tachyarrhythmias [17]
**Pompe disease**		10% [18]		The short PR interval can normalize after ERT (in infantile disease) [18]
**PRKAG2 syndrome**		68% [20]		Risk of lethal arrhythmias and SCD (high-degree AV block or fast conduction of atrial tachyarrhythmias) [20]
**AFD**	Stored glycosphingolipids increasing conduction velocity	15–40% [98]	Early [93]	The short PR interval can normalize after ERT [15]
**Mitochondrial disease**	Proliferation of mitochondria altering cardiomyocyte functioning	22% [21]		PM implantation may be considered in KSS
**Atrial fibrillation**				
**HCM**	Atrial enlargement and fibrosis	17–30% [24, 25]		Higher risk of all-cause mortality, cardiac death, SCD and stroke-related death [23, 26, 27]
**DCM**		36–76% [23, 24, 28–32]		
**ACM**	Desmosomal dysfunction and RA enlargement or dysfunction	9–30% [33, 34]		AF predicts worse outcomes in patients with right-sided ACM [36]
**LVNC**		1–29% [23, 24, 37, 38]	Lower incidence in children	AF is associated with LVNC severity and predicts survival [39]
**CA**	Amyloid infiltration promoting atrial fibrosis and dilation	15–44% [41, 42] Higher prevalence in ATTR- than AL-CA		AF does not seem to predict a worse outcome [44, 45]
**Q waves**				
**HCM**	Myocardial fibrosis and septal hypertrophy displacing the septal electrical vector	18–53% [46, 53]	Early	May precede the increase in LV mass by several years, are less common in patients with biventricular hypertrophy, and help differentiate HCM from the athlete’s heart
**CA**	Accumulation of amyloid and fibrosis	25–47% in AL-CA 18% in ATTR-CA [49, 50]		Independently predict death in patients with AL-CA [99]
**Idiopathic DCM**	Vector displacement due to LV dilation and transmural fibrosis	10–25% [3, 48]		
**Dystrophin cardiomyopathy**	Scarring of the posterolateral region of the LV	21% (DMD) [100], 42% (BMD) [101]		
**QRS complex: high voltages**				
**HCM**	Cardiomyocyte hypertrophy	41–60% [46, 53]		Scores including QRS amplitude were proposed to predict SCD [55] Mavacamten may relieve ECG signs of LVH
**DCM**	LVH	17–69% [56, 57]		Unclear prognostic relevance
**CA**	LV apex spared by amyloid accumulation	Up to 25% in ATTR-CA [58]		No relationship with outcome [49]
**Pompe disease, Danon disease, FD**	Accumulation of glycosphingolipids	6–10% (less common in Pompe than in Danon and AFD) [59]		
**QRS complex: low voltages**				
**HCM**	Extensive fibrosis	< 3% [61]	End-stage	Higher risk of SCD [61]
**DCM**	Loss of vital myocardium and diffuse LV fibrosis	3–6% [3, 63]		Higher risk of death or heart transplantation, SCD, or life-threatening ventricular arrhythmias [3]
**ACM**	Fibro-fatty replacement	41% with LV involvement 17% without LV involvement [63]	Advanced	Higher risk of life-threatening ventricular arrhythmias and SCD [67]
**CA**	Amyloid and fibrosis	46–70% (higher in AL-CA) [68, 69]		Higher risk of CV death [69]
**QRS fragmentation and epsilon wave**				
**HCM**	Myocardial disarray, interstitial fibrosis, conduction impairment	75% [73]		Higher risk of ventricular tachyarrhythmias and SCD (++ in 3 coronary artery territories) [73]
**DCM**	Myocardial scar	23–26% [74]		Higher risk of ventricular tachycardias and all-cause death [74]
**Cardiac sarcoidosis**	Myocardial granulomas	50% [102]	Early	Association with LGE presence [102]
**ACM**	Fibro-fatty replacement in the subepicardial region of the RV free wall	85% [103]		QRS fragmentation: higher risk of VT, VF, and appropriate ICD discharges [78]. Epsilon waves in aVR: higher risk of HF hospitalization, HF-related death, SCD, and heart transplantation [76]
**RBBB**				
**HCM**		4–5% [79]		One-third of patients develop complete heart block after myectomy [79]
**AFD**		22% [14]		
**DCM**		7% [80]		Predictor of all-cause mortality [80]
**ACM**		19 [82] to 32% [81] in ARVC		
**LBBB**				
**HCM**		2% [83], 22% in end-stage HCM [84], up to 40% after septal myectomy [85]		LBBB after surgery not associated with worse outcome [79]
**DCM**	Degeneration or fibrosis of the conduction system	25–30% [87]		No worse survival in patients with DCM and LVEF 36–50% [87] Shorter survival in patients with idiopathic DCM developing LBBB [88]
**ST segment and T waves**				
**HCM**	- Epicardial cardiomyocytes depolarize and repolarize later than endocardial cardiomyocytes - ST-segment elevation may signal ventricular aneurysm		Early	Negative T waves in V3–V6 becoming positive may denote the development of an apical aneurysm STEMI patterns associated with higher risk of SCD [61]
**DCM**		T wave inversion in 62% of patients with *FLNC* mutations [104]		
**Right-side ACM**	Fibro-fatty infiltration in the subepicardium			- Negative T wave strongly related to disease presence - ST segment elevation with negative T wave in V1–V2 related to advanced transmural RV involvement [92]
**CA**	Amyloid infiltration			
**AFD**				Asymmetrical negative T waves and ST-T segment depression or elevation in inferolateral leads related to fibrosis
**Mitochondrial diseases**		Asymmetrical negative T waves in 50% [21]		

*ACM* arrhythmogenic cardiomyopathy, *AF* atrial fibrillation, *AFD* Anderson-Fabry disease, *AL* light chain amyloidosis, *ATTR* transthyretin amyloidosis, *AV* atrioventricular, *BBB* bundle branch block, *BMD* Becker muscular dystrophy, *CA* cardiac amyloidosis, *DCM* dilated cardiomyopathy, *DM* myotonic dystrophy, *DMD* Duchenne muscular dystrophy, *DMD* dystrophin gene, *ECG* electrocardiogram, *ERT* enzyme replacement therapy, *FLNC* filamin-C gene, *HCM* hypertrophic cardiomyopathy, *HF* heart failure, *ICD* implantable cardioverter-defibrillator, *KSS* Kearns-Sayre syndrome, *LA* left atrium, *LBBB* left bundle branch block, *LGE* late gadolinium enhancement, *LMNA* lamin A/C, *LV* left ventricle, *LVH* left ventricular hypertrophy, *LVNC* left ventricular non-compaction cardiomyopathy, *M* male, *PM* pacemaker, *PRKAG2* protein kinase AMP-activated non-catalytic subunit gamma 2, *QTc* corrected QT, *RA* right atrium, *RBBB* right bundle branch block, *RV* right ventricle, *SCD* sudden cardiac death, *SCN5A* sodium voltage-gated channel alpha subunit 5 gene, *v* variant, *STEMI* ST elevation myocardial infarction, *VF* ventricular fibrillation, *VT* ventricular tachycardia, *wt* wild-type

### Supplementary Information

Below is the link to the electronic supplementary material.

Supplementary file1 (DOCX 1605 KB)

## Data Availability

This is not applicable.
