# Supplementary material for: Electrocardiographic abnormalities in patients with cardiomyopathies
Source: Heart Fail Rev. 2023 Oct 17;29(1):151–64. doi: 10.1007/s10741-023-10358-7 (PMC10904564; doi:10.1007/s10741-023-10358-7)
Supplement: Supplementary file 1 — Supplementary file1 (DOCX 1605 KB) [file 10741_2023_10358_MOESM1_ESM.docx]

**Supplemental material**

**Electrocardiographic abnormalities in patients with cardiomyopathies**

Alberto Aimo^1,2^, Agnese Milandri^3^, Andrea Barison^1,2^, Andrea Pezzato^4^, Paolo Morfino^1^, Giuseppe Vergaro^1,2^, Marco Merlo^4^, Alessia Argirò^5^, Iacopo Olivotto^6^, Michele Emdin^1,2^, Gherardo Finocchiaro^7,8^, Gianfranco Sinagra^4^, Perry Elliott^9^, Claudio Rapezzi^9┼^

1. Interdisciplinary Center for Health Sciences, Scuola Superiore Sant’Anna, Pisa, Italy; 2. Cardiology Division, Fondazione Toscana Gabriele Monasterio, Pisa, Italy; 3. Cardiology Unit, Bentivoglio Hospital, Bologna, Italy; 4. Center for Diagnosis and Management of Cardiomyopathies; Cardiothoracovascular Department Azienda Sanitaria Universitaria Giuliano Isontina (ASUGI) and University of Trieste, Italy; 5. Careggi University Hospital, Florence, Italy; 6. Department of Experimental and Clinical Medicine, University of Florence, Meyer Children Hospital Florence, Italy; 7. Royal Brompton and Harefield Hospital, London, UK; 8. St George’s University of London, UK; 9. UCL Centre for Heart Muscle Disease and lead of the Inherited Cardiovascular Disease Unit, Bart's Heart Centre, London, UK; 10. Cardiology Centre, University of Ferrara, Italy. ┼ deceased.


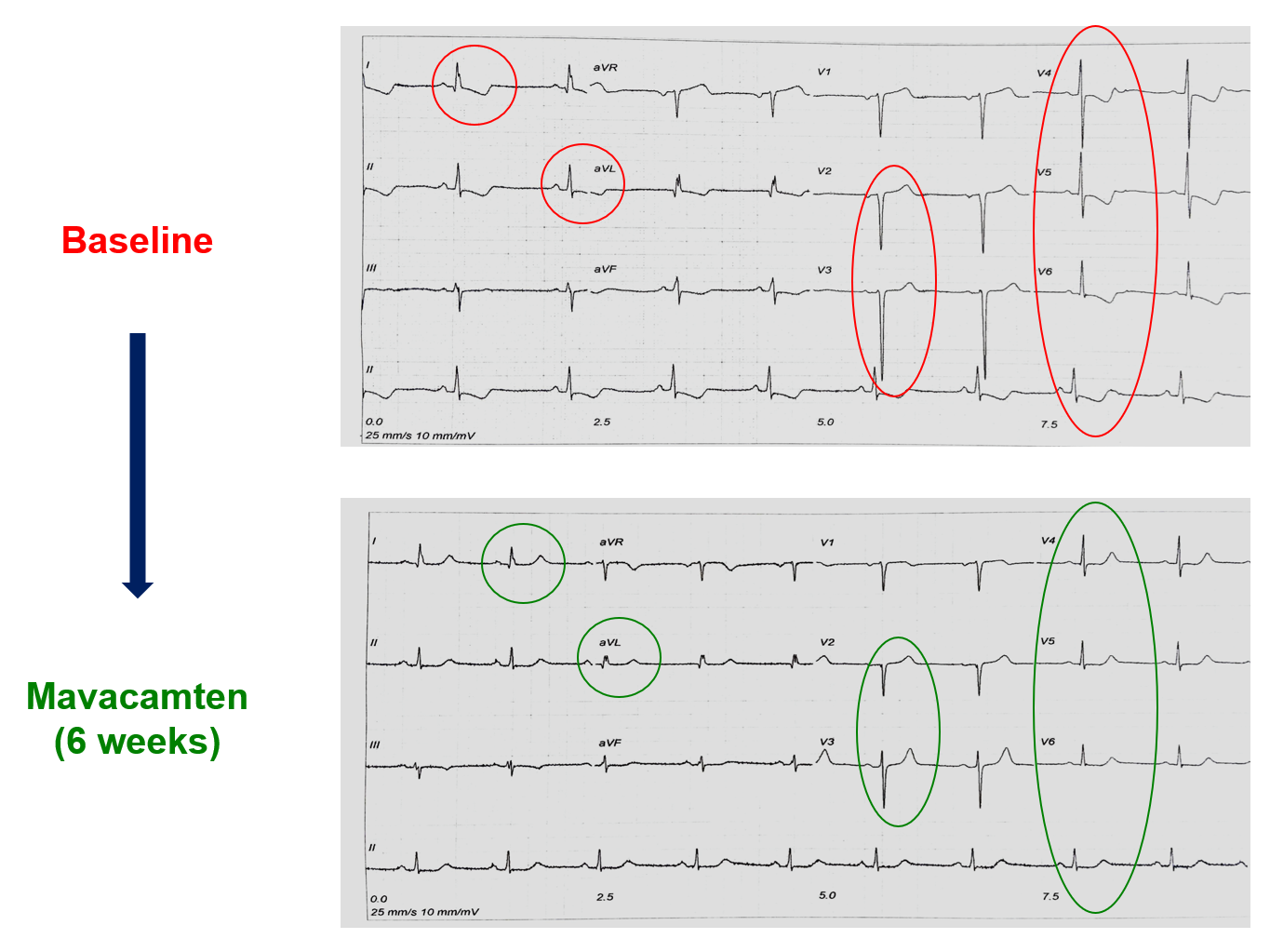


**Supplemental Figure 1. Regression of electrocardiographic abnormalities in a 50-year-old patient with hypertrophic cardiomyopathy following therapy with mavacamten for 6 weeks.**

Courtesy of Iacopo Olivotto.


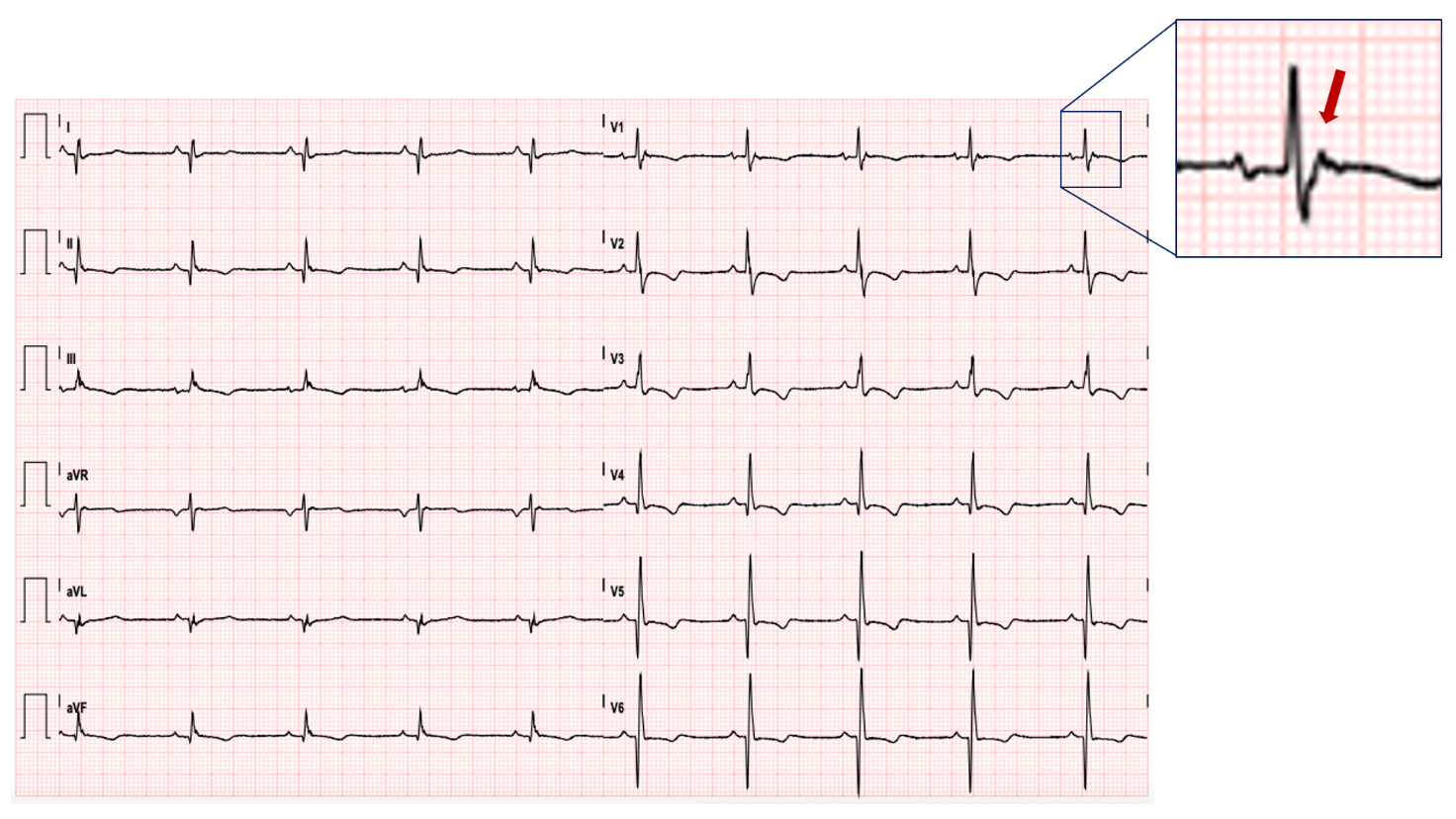


**Supplemental Figure 2. The epsilon wave.**
